# Single-cell RNA sequencing elucidates cellular plasticity in esophageal small cell carcinoma following chemotherapy treatment

**DOI:** 10.3389/fgene.2024.1477705

**Published:** 2025-01-09

**Authors:** Qinkai Zhang, Ziyu Gao, Ru Qiu, Jizhao Cao, Chunxiao Zhang, Wei Qin, Meiling Yang, Xinyue Wang, Ciqiu Yang, Jie Li, Dongyang Yang

**Affiliations:** ^1^ Center for Stem Cell Biology and Tissue Engineering, Key Laboratory for Stem Cells and Tissue Engineering, Ministry of Education, Sun Yat-sen University, Guangzhou, China; ^2^ Department of Breast and Thyroid Surgery, Guangzhou Women and Children’s Medical Center, Guangzhou, Guangdong, China; ^3^ Department of Thyroid Surgery, The First Affiliated Hospital of Sun Yat-sen University, Guangzhou, Guangdong, China; ^4^ School of Laboratory Medicine, Guangzhou Health Science College, Guangzhou, China; ^5^ Medical College, Jiaying University, Meizhou, China; ^6^ Guangdong Cardiovascular Institute, Guangdong Provincial People’s Hospital, Guangdong Academy of Medical Sciences, Guangzhou, China; ^7^ Medical Research Institute, Guangdong Provincial People’s Hospital (Guangdong Academy of Medical Sciences), Southern Medical University, Guangzhou, China; ^8^ Medical Oncology, Guangdong Provincial People’s Hospital (Guangdong Academy of Medical Sciences), Southern Medical University, Guangzhou, China

**Keywords:** small cell carcinoma of the esophagus, single-cell RNA sequencing, chemotherapy, tumor microenvironment, cell plasticity

## Abstract

Small cell carcinoma of the esophagus (SCCE) is a rare and aggressively progressing malignancy that presents considerable clinical challenges.Although chemotherapy can effectively manage symptoms during the earlystages of SCCE, its long-term effectiveness is notably limited, with theunderlying mechanisms remaining largely undefined. In this study, weemployed single-cell RNA sequencing (scRNA-seq) to analyze SCCE samplesfrom a single patient both before and after chemotherapy treatment. Our analysisrevealed significant cellular plasticity and alterations in the tumormicroenvironment’s cellular composition. Notably, we observed an increase intumor cell diversity coupled with reductions in T cells, B cells, and myeloid-likecells. The pre-treatment samples predominantly featured carcinoma cells in amiddle transitional state, while post-treatment samples exhibited an expandedpresence of cells in terminal, initial-to-terminal (IniTerm), and universally alteredstates. Further analysis highlighted dynamic interactions between tumor cells andimmune cells, with significant changes detected in key signaling pathways, suchas TIGIT-PVR and MDK-SDC4. This study elucidates the complex dynamics of cellplasticity in SCCE following chemotherapy, providing new insights and identifyingpotential therapeutic targets to enhance treatment efficacy.

## Introduction

Esophageal cancer is a leading cause of morbidity and mortality among gastrointestinal malignancies globally (Lagergren et al., 2017; GBD 2017 Oesophageal Cancer Collaborators (2020); Yang et al., 2024). Small cell carcinoma of the esophagus (SCCE), a particularly virulent subtype, represents only 0.4%–2.8% of all esophageal cancer cases (Wang et al., 2018; Ji et al., 2020). SCCE is distinguished by its rapid progression and pronounced predisposition for early metastasis, frequently presenting with extensive metastatic spread at diagnosis, which significantly restricts treatment options and outcomes. The median survival time post-diagnosis is merely 8–13 months, indicative of a severe prognosis for affected individuals (Chen et al., 2011). Furthermore, survival rates are bleak, with the majority of patients dying within 2 years (Chen et al., 2011; Ji et al., 2020; Zemerly et al., 2023). Treatment protocols for SCCE are largely adapted from those established for small cell lung cancer (SCLC) (Wang et al., 2019) and although initial chemotherapy responses are initially promising, they are generally fleeting, with rapid recurrence as a frequent consequence. The reduced efficacy of follow-up treatments exacerbates the challenge of managing SCCE, underscoring the urgent need for advanced understanding and development of more effective therapeutic strategies (Bennouna et al., 2000; Pantvaidya et al., 2002; Wu et al., 2024).

Cancer cell plasticity, defined as the capacity of cancer cells to alter their phenotypic traits and behaviors in response to environmental stimuli, plays a pivotal role in oncogenesis and cancer progression (Marjanovic et al., 2020; Salcher et al., 2022). Chemotherapy, a key therapeutic intervention, can induce marked phenotypic changes in cancer cells, including alterations in morphology, size, and surface marker expression (Pomeroy et al., 2022; Lyman et al., 2014). These changes often lead to a transition towards more stem-like states, which can enhance resistance to therapeutic agents and facilitate metastatic spread (Han et al., 2022). Despite considerable research, the specific impact of chemotherapy on the plasticity of ESCC cells has not been sufficiently explored. Additionally, the tumor microenvironment (TME) significantly influences cancer cell plasticity. Post-chemotherapy, modifications in both the cellular composition and the extracellular matrix of the TME can convey signals that promote survival, adaptation, and invasiveness of the remaining cancer cells (Wang et al., 2019; Hinshaw and Shevde, 2019; Bader et al., 2020). Recent studies have demonstrated that chemotherapy can trigger significant immunological shifts within the TME, such as the proliferation of regulatory T cells and an increase in the expression of immune checkpoint molecules like TIGIT and PD-L1 (20–22). These findings underscore the dual role of the TME in both mediating therapeutic responses and facilitating resistance. However, the precise characteristics and dynamics of the TME in SCCE remain poorly understood.

Recent advancements in single-cell transcriptomics have substantially enhanced our understanding of cancer’s cellular heterogeneity, the oncogenic mechanisms that drive its progression, and the immunosuppressive landscape of the TME (Krämer et al., 2020). For instance, single-cell analyses have revealed previously unrecognized tumor cell states linked to therapy resistance in breast cancer and have elucidated complex interactions between cancer cells and immune cells within the TME (Fang et al., 2024). In this study, we explore the cellular dynamics within esophageal small cell carcinoma (SCCE) following chemotherapy with irinotecan and cisplatin. Our results demonstrate significant plasticity in SCCE cells, marked by notable reductions in T cells, B cells, and myeloid-like B cells after treatment. Additionally, we detail the intricate interactions between tumor cells and the immune components of the TME in SCCE, thereby deepening our understanding of the tumor’s adaptive responses to chemotherapeutic interventions.

## Methods and materials

### Sample collection and preparation

Esophageal carcinoma specimens, comprising both pre-treatment and post-treatment paired samples, were procured from a 66-year-old male diagnosed with small cell carcinoma of the esophagus (SCCE). The diagnosis was initially established via esophagogastroduodenoscopy, which identified a significant lesion located between 24 and 28 cm from the incisors on the anterior side of the esophagus. The lesion displayed cauliflower-like erosion and spanned approximately one-third of the esophageal circumference. Histopathological examination confirmed the lesion as small cell carcinoma, staged as T4N + M0, consistent with Stage III disease. All procedures performed in the study were approved by the Ethics Committee of Guangdong Provincial People’s Hospital (Ethics Approval Number: KY-Z-2021-507-02).

## Treatment regimen and response evaluation

From 19 September 2022, to 17 January 2023, the patient received five cycles of chemotherapy comprising irinotecan and cisplatin. A CT scan conducted on 31 October 2022, demonstrated a Partial Response (PR) to the treatment. On 15 February 2023, the treatment strategy was augmented to include concurrent chemoradiation therapy, utilizing volumetric modulated arc therapy (VMAT) which delivered a total radiation dose of 41 Gy across 23 fractions to the planning clinical target volume (PCTV), with an additional 20 Gy administered over 10 fractions targeting the planning gross tumor volume (PGTV). The chemoradiation regimen was complemented by two cycles of etoposide and lobaplatin.

Continued surveillance through a CT scan on 12 May 2023, confirmed the maintenance of PR. Subsequently, from 13 May 2023, to 27 September 2023, the patient underwent seven cycles of teriparatide monoclonal antibody therapy. Despite these extensive therapeutic efforts, a CT scan on 27 September 2023, indicated Progressive Disease (PD). The patient’s progression-free survival (PFS) was recorded at 11 months.

Despite the initial benefits from the comprehensive treatment strategy, the patient’s health deteriorated rapidly following disease progression, resulting in an overall survival (OS) of only 14 months.

### Immunofluorescence staining

Formalin-fixed, paraffin-embedded (FFPE) tissue sections underwent antigen retrieval using citrate buffer (pH 6) in a microwave oven for 30 min following deparaffinization. Subsequent to rinsing with phosphate-buffered saline (PBS), the sections were permeabilized and blocked with a solution of Tris-buffered saline (TBS) containing 0.3% Triton X-100% and 5% donkey serum (D-TBST) for 1 h at room temperature. The tissue sections were then incubated overnight at 4°C with primary antibodies diluted in D-TBST solution.

Post-primary antibody incubation, the sections were washed thrice with TBS containing 0.05% Tween-20, followed by incubation with the secondary antibody and Hoechst stain (1:1,000) diluted in D-TBST for 1.5 h at room temperature. Primary antibodies used included CD3 (ab16669) and CD68 (ab955).

## scRNA-seq library preparation and sequencing

Single-cell suspensions were derived from the collected samples through a dual process of enzymatic digestion and mechanical dissociation, meticulously designed to preserve cellular integrity and viability. These prepared cells were subsequently processed utilizing the 10x Genomics Chromium Single Cell 3′platform, adhering stringently to the manufacturer’s protocols. The resulting libraries were sequenced using an Illumina NovaSeq 6000 system, generating high-throughput, paired-end reads of 150 base pairs each.

### Initial quality control and data integration

The scRNA-seq datasets from the pre-treatment and post-treatment samples were initially uploaded for analysis. We conducted rigorous quality control using the Seurat package (version 5.0.3) within the R programming environment. This phase involved stringent filtering to exclude low-quality cells and genes from the datasets. The quality control criteria included: (Lagergren et al., 2017): exclusion of cells with fewer than 200 or more than 10,000 detected genes (nFeature_RNA) to eliminate low-complexity cells and potential multiplets; (GBD 2017 Oesophageal Cancer Collaborators, 2020); removal of cells with mitochondrial gene content exceeding 20% (percent.mt) or those with low unique molecular identifier (UMI) counts (below 200) to eradicate low-quality or stressed cells. Data quality was further evaluated using scatterplots (nCount_RNA vs. percent. mt, and nCount_RNA vs. nFeature_RNA) and violin plots of nFeature_RNA, nCount_RNA, and percent. mt.

Following the filtering process, datasets were normalized employing the “LogNormalize” method with a scale factor of 10,000. Highly variable genes were identified using the variance stabilizing transformation (VST) method, selecting the top 5,000 features. For comparative analyses, the datasets were integrated using anchor-based methods (FindIntegrationAnchors and IntegrateData) across 30 dimensions.

### Data normalization and integration

Data normalization across the datasets was performed using the “LogNormalize” method. To effectively integrate the pre-treatment and post-treatment datasets, integration anchors were established based on anchor genes identified within each dataset. Utilizing these anchors, the datasets were seamlessly integrated, with meticulous attention to correcting batch effects throughout this process.

### Dimensionality reduction and clustering

Dimensionality reduction of the integrated scRNA-seq data was achieved using Principal Component Analysis (PCA). The initial computation included the first 50 principal components. To ascertain the optimal number of components to retain for further analysis, we employed both the JackStraw method and the Elbow plot, ensuring robustness in subsequent analyses.

For visual representation of the reduced-dimensional data, we utilized two techniques: Uniform Manifold Approximation and Projection (UMAP) and t-distributed Stochastic Neighbor Embedding (t-SNE). UMAP calculations were specifically performed using the first 20 principal components, which provided a clear visualization of the underlying data structure.

Cell clustering was executed using the Louvain algorithm, as implemented in the Seurat package. To determine the most appropriate clustering resolutions, we systematically evaluated outcomes based on UMAP visualizations, clustree analysis, and marker gene expression heatmaps. The clustree method was employed to identify a suitable range of resolutions, while UMAP plots and heatmaps were used to validate biologically meaningful distinctions between clusters. Following this comprehensive assessment, a resolution of 1.1 was selected for the initial clustering, facilitating robust segregation of cell populations. This resolution allowed for the detailed clustering of cells, with cluster compositions in both pre-treatment and post-treatment samples analyzed and depicted using bar plots.

Additionally, specific cell populations—including epithelial cells, T cells, and myeloid cells—were isolated for sub-clustering to further explore cellular heterogeneities. The resolutions for sub-clustering were optimized as follows: 0.6 for epithelial and myeloid cells, and 1.4 for T cells, ensuring that distinct biological features and interactions within these populations were effectively captured.

### Cell type annotation

To accurately annotate cell types within the Small Cell Carcinoma of the Esophagus (SCCE) dataset, we compiled an extensive list of marker genes from reliable sources, including Cell Signaling Technology (https://www.cellsignal.com/pathways/immune-cell-markers-human), Abcam (https://www.abcam.com/primary-antibodies/immune-cell-markers-poster), and various scholarly publications. These marker genes were instrumental in categorizing cells into distinct types according to their gene expression profiles.

The expression levels of these cell type-specific markers were visualized using dot plots and violin plots to facilitate the validation of cell type annotations. These visualizations were generated using the “ggplot2” package (version 3.5.1) in R. Additionally, we quantified the proportions of each cell type in individual patient samples, using bar plots to display the relative percentages of different cell types in both pre-treatment and post-treatment samples.

For myeloid cells, where marker gene expression levels did not markedly differ among subtypes, cell type annotation was further refined based on the functions enriched within these subtypes. This refined approach allowed for a more nuanced understanding of myeloid cell diversity within the SCCE samples.

### InferCNV analysis

To identify and characterize genomic copy number variations (CNVs) within the epithelial cell populations of SCCE, we employed the inferCNV R package (version 1.18.1). This analytical tool is specifically designed to detect large-scale chromosomal alterations in single-cell RNA sequencing (scRNA-seq) data by assessing gene expression intensities across various cells. The CNV profiles generated by inferCNV offered pivotal insights into the genomic architecture of SCCE tumor cells, underscoring potential driver mutations and regions of genomic instability.

### Differential expression analysis

To pinpoint genes exhibiting significant changes in expression between pre-treatment and post-treatment samples, as well as among different cellular subgroups, we performed a differential expression analysis. In this analysis, genes were classified as significantly differentially expressed based on stringent criteria: an adjusted *p*-value of less than 0.05 and an average log fold change exceeding 0.25. These thresholds were set to ensure that the differences in gene expression observed were not only statistically significant but also biologically meaningful.

### Functional enrichment analysis

Gene Ontology (GO) enrichment analysis was conducted to explore the biological processes (BP), cellular components (CC), and molecular functions (MF) linked to genes significantly differentially expressed between pre-treatment and post-treatment samples of small cell carcinoma of the esophagus (SCCE), as well as among various cellular subgroups. GO terms that showed enrichment with a false discovery rate (FDR) less than 0.05 were deemed significant.

Concurrently, Kyoto Encyclopedia of Genes and Genomes (KEGG) pathway enrichment analysis was performed to identify crucial metabolic and signaling pathways affected by the differentially expressed genes (DEGs). Pathways with an FDR less than 0.05 were classified as significantly enriched. The results of these analyses were visualized to clearly delineate the roles of DEGs in various biological pathways, with a particular focus on those pathways that are relevant to SCCE drug resistance.

### Pseudotime analysis

To explore the dynamic changes in cell states and identify critical transition points during the treatment of SCCE, we employed pseudotime analysis using the Monocle package (version 2.30.1) in R. This analysis began with the construction of a cell trajectory dataset from normalized and integrated single-cell RNA sequencing (scRNA-seq) data. The gene expression matrix was refined to emphasize highly variable genes, which are essential for mapping cellular trajectories and understanding dynamic biological processes.

The DDRTree algorithm was utilized for dimensionality reduction and trajectory inference. To determine the start and end points of pseudotime, cells were grouped into specific clusters based on their expression profiles, and these clusters were annotated with biologically meaningful states. This systematic annotation provided a clear framework for ordering cells along the pseudotime trajectory. Throughout the pseudotime analysis, differential gene expression analysis was performed to identify genes that significantly altered their expression as cells transitioned through various states. This methodology yielded profound insights into the molecular mechanisms driving cellular transitions during SCCE treatment, enhancing our understanding of the disease’s progression and response to therapy.

### Cell-cell communication analysis

To investigate cell-cell communication within samples of SCCE, we utilized the “CellChat” package (version 1.6.1) in R. This tool is specifically designed to identify significant ligand-receptor interactions across various cell types, thereby elucidating the dynamics of intercellular signaling.

Through the use of CellChat, we pinpointed key ligand-receptor interactions and mapped these onto communication networks, thus revealing the intricate interactions both within and among different cell types. We employed a variety of visualization techniques, including network and circos plots, to illustrate the complexity and specificity of these intercellular signaling pathways. These visualizations greatly enhanced our understanding of the communication patterns that may impact disease progression and response to treatment in SCCE.

#### Gene set enrichment analysis (GSEA)

GSEA was conducted using the clusterProfiler package (version 4.10.1) in R to delineate the biological processes and signaling pathways implicated in SCCE. Key genes involved in cellular communication were identified and their fold changes computed using the “FindMarkers” function in the Seurat package. These genes were then ranked based on their log fold-change values.

Canonical pathways and GO biological processes were referenced from the Molecular Signatures Database (MSigDB). Enrichment scores for each gene set were calculated, with the significance of these scores determined through permutation tests. Pathways and processes with a *p*-value less than 0.05 were deemed significantly enriched. This analysis yielded critical insights into the biological mechanisms and pathways associated with drug resistance in SCCE, enhancing our understanding of disease pathology and identifying potential therapeutic targets.

#### Protein-protein interaction (PPI) network construction

We constructed a PPI network focusing on key ligands and receptors identified in our study of SCCE. The interactions were sourced from the STRING database (https://string-db.org/), which is a comprehensive resource for both known and predicted protein-protein interactions. The network was visualized using Cytoscape (version 3.10.2), a software tool specifically designed for complex network analysis and visualization.

This visualization of the PPI network facilitates an understanding of the interaction dynamics among proteins, providing key insights into the molecular mechanisms that may play critical roles in the pathogenesis and treatment response of SCCE. This approach not only enhances our understanding of the connectivity among proteins but also underscores potential therapeutic targets within the network.

### Statistical analysis

Statistical analyses for this study were performed using R software (version 4.3.3). The primary method for assessing differential expression was the Wilcoxon rank-sum test, with Bonferroni correction applied to adjust for multiple comparisons and control the family-wise error rate.

For the analysis of cell-cell interactions, significant interactions were identified based on a communication probability (*p*-value) of less than 0.05. Additionally, variations in cell type proportions across different subgroups were evaluated using Fisher’s exact test to ascertain their statistical significance.

In the pseudotime analysis conducted with Monocle, changes in gene expression along the pseudotime trajectory were assessed using the likelihood ratio test, which identifies statistically significant changes in gene expression corresponding to progression through different cellular states.

All statistical tests employed in this study were two-tailed, with a significance threshold set at a *p*-value of less than 0.05, unless specified otherwise. This rigorous analytical framework ensures that the findings are both reliable and statistically robust.

## Results

### Single-cell RNA sequencing reveals dynamic cellular remodeling in small cell carcinoma of the esophagus following treatment

**FIGURE 1:**
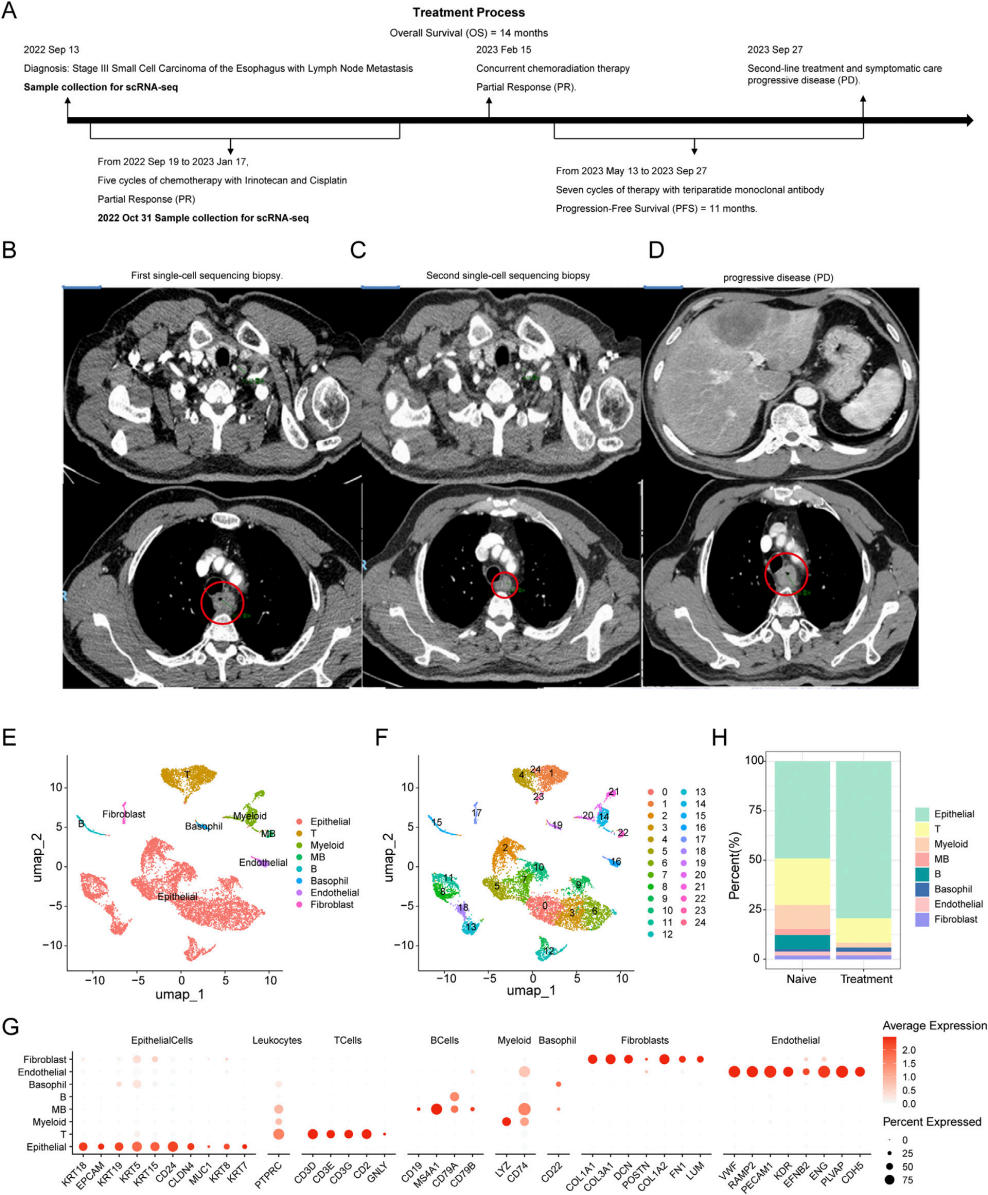
Comprehensive Analysis of Cellular Composition and Clustering in SCCE Across Treatment Regimens **(A)**. Timeline of treatments and scRNA-seq sampling for a 66-year-old male diagnosed with SCCE, detailing the sequence of therapeutic interventions and corresponding sampling points. **(B)** Computed tomography (CT) scan image at diagnosis, displaying a prominent lesion located between 24 and 28 cm from the incisors on the anterior esophagus, highlighted within a red circle. **(C)** CT scan image from 31 October 2022, following the completion of five chemotherapy cycles with irinotecan and cisplatin, showing a reduction in tumor size, indicated by the area within the red circle. **(D)** CT scan from 27 September 2023, showing progressive disease (PD) despite the patient undergoing concurrent chemoradiation therapy and multiple cycles of teriparatide monoclonal antibody therapy, with the tumor area highlighted within the red circle. **(E)** UMAP visualization of eight major cell types within the SCCE microenvironment, including epithelial cells, T cells, myeloid cells, myeloid-like B cells (MB), B cells, basophils, endothelial cells, and fibroblasts. **(F)** Results of clustering analysis using the Seurat package, identifying 25 distinct cellular clusters, each annotated based on the expression profiles of established marker genes. **(G)** Bar chart presenting a comparative quantitative analysis of cell type distributions before and after treatment, detailing the proportional changes in each cell type across naive and treated samples.

A 66-year-old male diagnosed with SCCE underwent a comprehensive treatment regimen, analyzed through scRNA-seq. The patient’s overall survival (OS) post-diagnosis was 14 months, with the timeline of treatment and sampling detailed in Figure 1A.

An esophagogastroduodenoscopy performed on 13 September 2022, revealed a raised, cauliflower-like erosive lesion located 24–28 cm from the incisors, predominantly on the anterior esophagus, affecting approximately one-third of the esophageal circumference (Figure 1B). Histopathological examination confirmed the diagnosis of SCCE, classified as T4N + M0, Stage III. Samples for scRNA-seq analysis were collected at this time. From 19 September 2022, to 17 January 2023, the patient received five cycles of chemotherapy with irinotecan and cisplatin. A follow-up CT scan on 31 October 2022, indicated a Partial Response (PR) (Figure 1C), prompting a second single-cell sequencing biopsy.

The treatment strategy was intensified on 15 February 2023, with the initiation of concurrent chemoradiation therapy. This regimen included volumetric modulated arc therapy (VMAT) delivering 41 Gy over 23 fractions to the planning clinical target volume (PCTV) and an additional 20 Gy over 10 fractions to the planning gross tumor volume (PGTV). Concurrently, two cycles of etoposide and lobaplatin were administered.

By 12 May 2023, a CT scan confirmed the maintenance of PR. From 13 May 2023, to 27 September 2023, the patient underwent seven cycles of teriparatide monoclonal antibody therapy. Despite these interventions, a CT scan on 27 September 2023, demonstrated progressive disease (PD) (Figure 1D), with a progression-free survival (PFS) documented at 11 months. Despite the initial benefits from this multimodal treatment approach, the disease’s subsequent progression led to rapid clinical deterioration.

scRNA-seq analysis of SCCE revealed a complex cellular architecture undergoing significant transformation post-treatment with irinotecan and cisplatin (Figure 1E). Using the Seurat package for clustering analysis, we identified 25 distinct cellular clusters within the esophageal carcinoma microenvironment (Figure 1F). These clusters were annotated based on the expression profiles of known marker genes (Figure 1G). Comparative analysis between pre-treatment (naive) and post-treatment (treated) samples revealed substantial shifts in cellular composition (Figure 1H). Immunofluorescence staining of primary SCCE samples revealed a marked prevalence of T cells and myeloid-like cells (Supplementary Figure S1). Notably, the proportion of epithelial cells increased following treatment, whereas the populations of T cells, B cells, and myeloid-like cells decreased.

### Transcriptional reprogramming of epithelial carcinoma cells in response to treatment in SCCE

**FIGURE 2:**
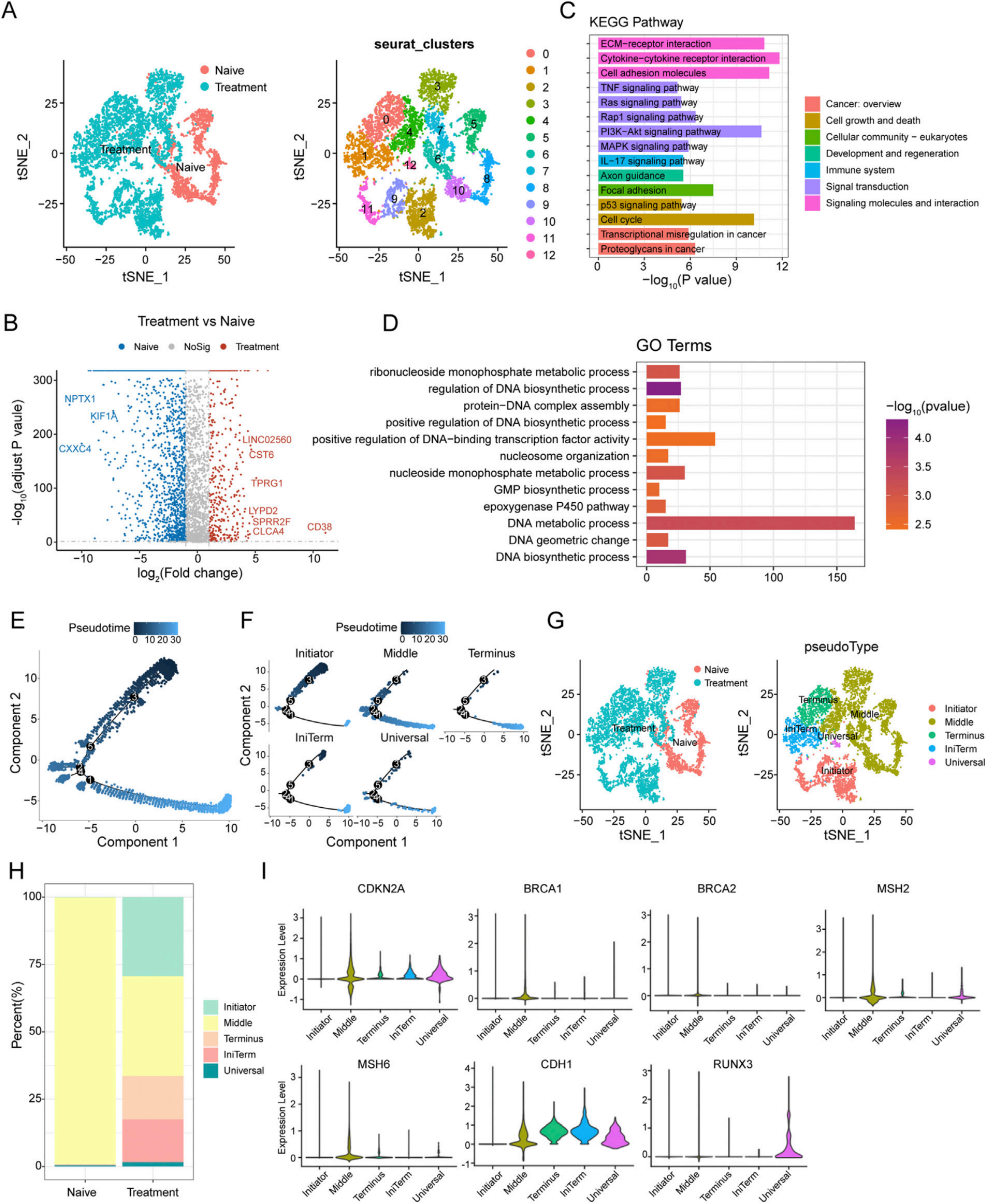
Epithelial Cell Dynamics and Pseudotime Analysis in SCCE Following Treatment **(A)** t-SNE analysis illustrating distinct clustering patterns of epithelial cells from SCCE, revealing variability in cellular states post-treatment. **(B)** Volcano plot displaying differentially expressed genes (DEGs) between naive and treated epithelial cells. Upregulated genes in treated cells such as LINC02560, CST6, TPRG1, LYPD2, SPRR2F, CLC4A, and CD38 are highlighted in red, while downregulated genes post-treatment, including NPTX1, KIF1A, and CXXC4, are marked in blue. **(C)** KEGG pathway enrichment analysis of DEGs, identifying significantly enriched signaling pathways crucial for tumor cell interactions within their microenvironment. This includes pathways such as extracellular matrix (ECM)-receptor interactions, cytokine-cytokine receptor interactions, cell adhesion molecules, TNF signaling, Ras signaling, and PI3K-Akt signaling, which are essential for immune modulation and cell survival. **(D)** GO analysis illustrating significant enrichment in terms related to the molecular biology of treated epithelial cells. Key processes highlighted include nucleoside monophosphate metabolic process, regulation of DNA biosynthetic process, protein-DNA complex assembly, and positive regulation of DNA-binding transcription factor activity. **(E)** Pseudotime trajectory depicting the progression of epithelial cell states from the Initiator state, through Middle, to the Terminus state. **(F)** Clustering analysis of epithelial cells identifying five distinct groups: Initiator, Middle, Terminus, IniTerm (cells transitioning from initial to terminal states), and Universal (cells exhibiting characteristics of all stages). **(G)** t-SNE plots showcasing the segregation of these clusters in both naive and treatment groups. **(H)** Variations in the distribution of these clusters between naive and treatment groups, with naive samples predominantly concentrated in the Middle state, while treatment samples show an increase in cells classified as Terminus, IniTerm, and Universal states. **(I)** Violin plots illustrating expression patterns of key tumor suppressor genes across pseudotime clusters, such as CDKN2A, CDH1, BRCA1, BRCA2, MSH2, MSH6, and RUNX3. These plots indicate the significant roles of these genes in cellular state transitions and responses to treatment.

T-distributed Stochastic Neighbor Embedding (t-SNE) analysis of epithelial cells from SCCE samples demonstrated distinct clustering patterns between naive (pre-treatment) and treated groups (Figure 2A), indicating significant treatment-induced alterations in cellular states. Further genomic analysis using inferCNV confirmed prevalent mutations in these SCCE epithelial cells, substantiating their tumor cell classification.

Differential expression analysis between naive and treated carcinoma epithelial cells identified several significantly differentially expressed genes (DEGs). A volcano plot analysis highlighted the upregulation of genes such as LINC02560, CST6, TPRG1, LYPD2, SPRR2F, CLC4A, and CD38 in treated cells, while genes like NPTX1, KIF1A, and CXXC4 were notably downregulated (Figure 2B). KEGG pathway enrichment analysis of these DEGs revealed significant enrichment in pathways pivotal for tumor cell interactions with the microenvironment, including extracellular matrix (ECM)-receptor interactions, cytokine-cytokine receptor interactions, cell adhesion molecules, TNF signaling, Ras signaling, and PI3K-Akt signaling pathways (Figure 2C). GO term analysis showed significant enrichment in terms associated with nucleoside monophosphate metabolic processes, regulation of DNA biosynthetic processes, protein-DNA complex assembly, and positive regulation of DNA-binding transcription factor activity (Figure 2D).

Pseudotime analysis was utilized to explore cell plasticity in epithelial cell states during SCCE treatment. This analysis delineated a pseudotime trajectory depicting a continuous progression from an initial state (Initiator), through intermediate states (Middle), to a terminal state (Terminus) (Figure 2E). Clustering of epithelial cells along this trajectory identified five distinct clusters: Initiator, Middle, Terminus, IniTerm (cells transitioning from initial to terminal states), and Universal (cells exhibiting characteristics of all stages) (Figure 2F). t-SNE plots confirmed the distinct segregation of these clusters in both naive and treated groups (Figure 2G). Significant variations in the distribution of these clusters between naive and treated samples were observed (Figure 2H), with the naive group predominantly displaying a concentration of cells in the Middle state, while the treatment group showed an increase in cells within the Terminus, IniTerm, and Universal states, suggesting treatment-induced shifts in cell plasticity.

Additionally, the expression of key tumor suppressor genes was analyzed to elucidate their roles in cellular state transitions and responses to treatment. Violin plots demonstrated distinct expression patterns across different pseudotime clusters (Figure 2I), with genes such as CDKN2A and CDH1 showing high expression in the Middle and Terminus states, whereas BRCA1, BRCA2, MSH2, MSH6, and RUNX3 exhibited variable expression, underscoring their differential roles in regulating cell plasticity.

### T Cell diversity and dynamics in response to treatment in SCCE

**FIGURE 3:**
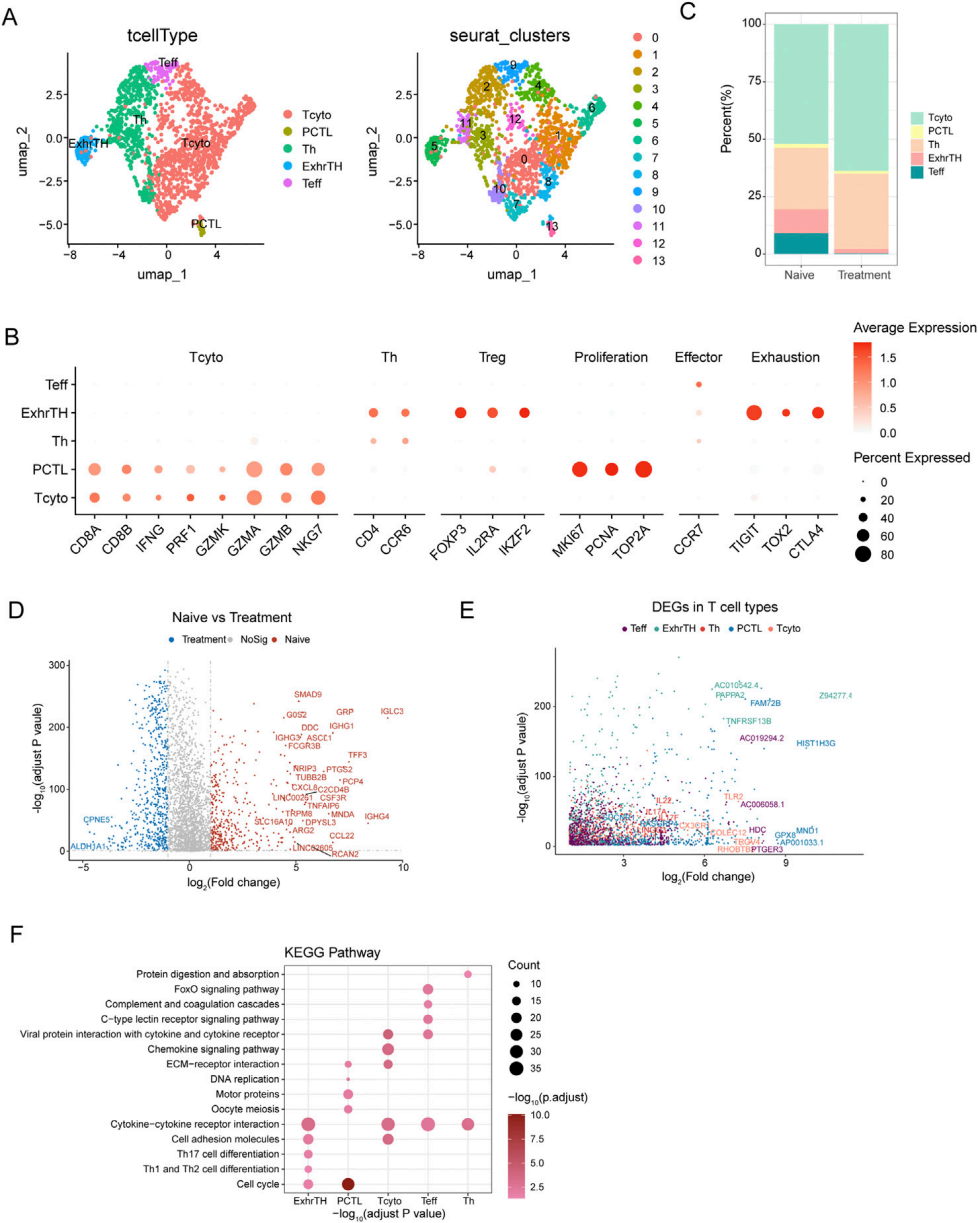
UMAP Analysis and Differential Gene Expression in T Cell Subtypes within Esophageal Carcinoma Samples **(A)** UMAP visualization identifying five major T cell subtypes within esophageal carcinoma samples, including cytotoxic T cells (Tcyto), primary cytotoxic T cells (PCTL), helper T cells (Th), exhausted T helper cells (ExhrTH), and effector T cells (Teff), with associated marker gene expression levels depicted. **(B)** Results of clustering analysis displaying six distinct cellular clusters of T cells identified using the Seurat package. **(C)** Comparative analysis of T cell subtype composition between naive and treatment samples, demonstrating increases in cytotoxic and helper T cells and decreases in exhausted T helper and effector T cells following treatment. **(D)** Differential gene expression analysis between naive and treated T cells, highlighting genes such as IGHG1, IGHA1, and IGKC, which were upregulated, and ALDH1A1 and IGFBP3, which were downregulated. **(E)** Analysis of gene expression patterns across T cell subtypes, noting upregulation of GZMB and PRF1 in Teff cells and increased expression of TIGIT and TOX2 in ExhrTH cells. **(F)** KEGG pathway enrichment analysis of differentially expressed genes, emphasizing significant pathways including cytokine-cytokine receptor interaction, cell adhesion molecules, extracellular matrix (ECM)-receptor interaction, and Th1 and Th2 cell differentiation. These pathways are crucial for T cell functionality and the immune response within the tumor environment.

Uniform Manifold Approximation and Projection (UMAP) analysis was utilized to investigate the heterogeneity of T cells within small cell carcinoma of the esophagus (SCCE) (Figure 3A). This analysis identified five major T cell subtypes: cytotoxic T cells (Tcyto), primary cytotoxic T cells (PCTL), helper T cells (Th), exhausted T helper cells (ExhrTH), and effector T cells (Teff), with their respective marker gene expression levels depicted in Figure 3B. Notably, substantial variations in the composition of these T cell subtypes were observed when comparing naive and post-treatment samples. Post-treatment, there was a significant increase in the populations of cytotoxic and helper T cells, while the numbers of exhausted T helper cells and effector T cells diminished (Figure 3C).

Differential expression analysis between naive and treated T cells revealed several significant differentially expressed genes (DEGs). For instance, genes such as IGHG1, IGHA1, and IGKC were upregulated in treated T cells, whereas genes like ALDH1A1 and IGFBP3 were downregulated, as detailed in Figure 3D. Further exploration of gene expression across different T cell subtypes revealed distinct patterns: Teff cells exhibited upregulation of GZMB and PRF1, while ExhrTH cells showed increased expression of TIGIT and TOX2 (Figure 3E).

KEGG pathway enrichment analysis of these DEGs highlighted significant pathways essential for T cell functionality, immune response, and their interactions with the tumor environment. These pathways included cytokine-cytokine receptor interaction, cell adhesion molecules, extracellular matrix (ECM)-receptor interaction, and Th1 and Th2 cell differentiation (Figure 3F).

### Dynamic interaction networks between tumor cells and T Cells in SCCE post-treatment

**FIGURE 4:**
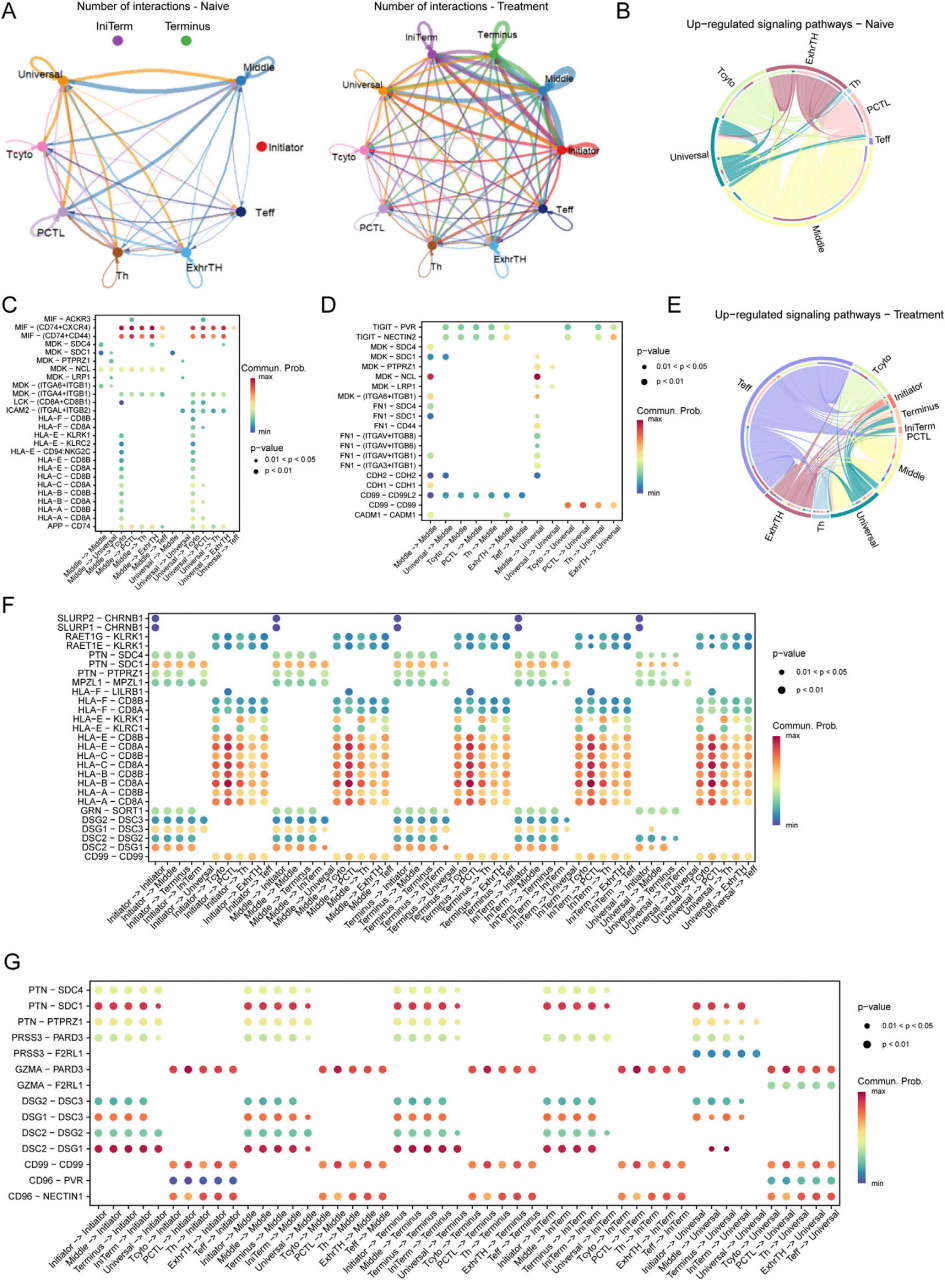
Interaction Networks and Signaling Pathways Between Tumor Cells and T Cells in the Esophageal Carcinoma Microenvironment Post-Treatment **(A)** Illustration of dynamic changes in cell-cell interactions between tumor cells (Initiator and Universal) and various T cell subtypes across naive and treated samples, demonstrating a marked increase in interactions following treatment. **(E–G)** Pathway analysis in treated samples, illustrating upregulated pathways: **(E)** The TIGIT-PVR pathway, indicative of enhanced immune checkpoint activity. **(F)** The MDK-SDC4 pathway, associated with immune checkpoint regulation. **(G)** The FN1-ITGA6/ITGB1 pathway, involved in increased cell adhesion and extracellular matrix remodeling.

Analysis of interaction networks between tumor cells and T cells within the esophageal carcinoma microenvironment revealed dynamic alterations following treatment (Figure 4A). In naive samples, interactions were predominantly observed between Initiator and Universal tumor cells and various T cell subtypes. Post-treatment, these interactions significantly increased, particularly involving Initiator cells and multiple T cell subtypes, underscoring substantial treatment-induced shifts in cell-cell communication.

Pathway analysis revealed distinct differences in signaling pathways between naive and treated conditions. In naive cells, critical pathways such as MIF-CD74/CXCR4, MDK-SDC1, and HLA-E-CD8A were predominant, playing pivotal roles in immune modulation and tumor cell survival (Figures 4B–D). Conversely, in treated cells, pathways including TIGIT-PVR, MDK-SDC4, and FN1-ITGA6/ITGB1 were upregulated, indicating enhanced immune checkpoint activity and increased cell adhesion (Figures 4E–G).

In the naive microenvironment, interactions involving Middle and Universal tumor cells were enriched in immune-related pathways such as MIF-CD74/CXCR4 and HLA-E-CD8A, suggesting a potentially immune-suppressive landscape (Figure 4C). Post-treatment, the interaction profile shifted to include pathways such as TIGIT-PVR and FN1-ITGA6/ITGB1, reflecting enhanced immune checkpoint regulation and extracellular matrix remodeling (Figure 4F).

Moreover, in naive samples, significant interactions were concentrated in pathways like MIF-CD74, MDK-SDC1, and HLA-E-CD8A, aligning with mechanisms of immune suppression and tumor cell survival. In contrast, treated samples exhibited prominent interactions involving Middle and Universal tumor cells through pathways such as TIGIT-PVR, MDK-SDC4, and FN1-ITGA6/ITGB1, indicative of an adaptive tumor response characterized by increased immune checkpoint activity and extracellular matrix remodeling (Figure 4G).

### Dissecting myeloid cell heterogeneity and dynamics in SCCE

**FIGURE 5:**
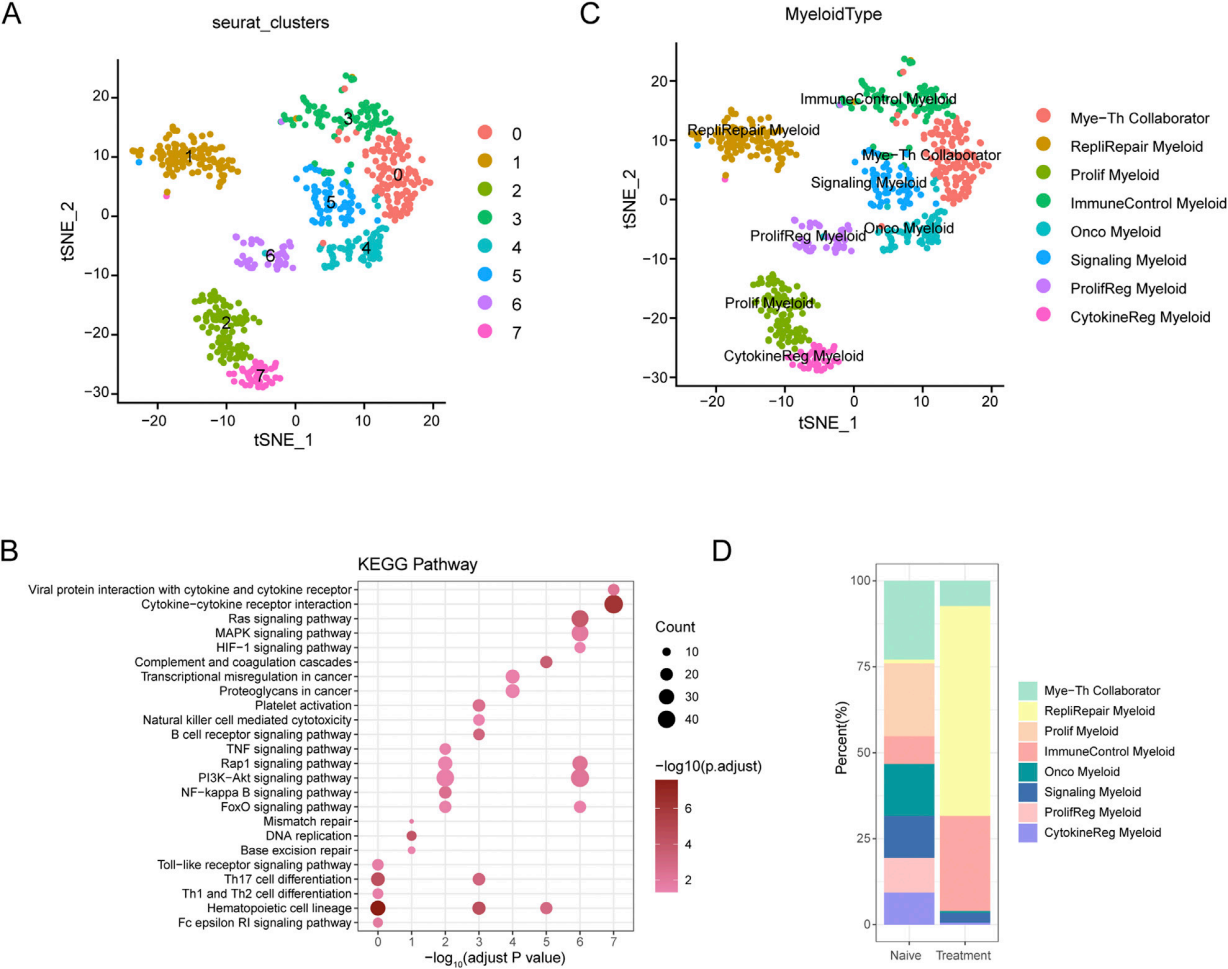
t-SNE Analysis and Pathway Enrichment in Myeloid Cells from SCCE **(A)** t-SNE plots illustrating eight distinct myeloid cell subtypes identified within SCCE samples: Myeloid-T helper collaborator (Mye-Th Collaborator), Replication-repair myeloid (RepliRepair Myeloid), Proliferation myeloid (Prolif Myeloid), Immune control myeloid (ImmuneControl Myeloid), Oncogenic myeloid (Onco Myeloid), Signaling myeloid (Signaling Myeloid), Proliferation regulatory myeloid (ProlifReg Myeloid), and Cytokine regulatory myeloid (CytokineReg Myeloid). **(B)** KEGG pathway enrichment analysis for the identified myeloid cell clusters, emphasizing critical pathways such as cytokine-cytokine receptor interaction, Ras signaling, PI3K-Akt signaling, and NF-kappa B signaling. These pathways are vital for myeloid cell functionality and their interactions within the tumor microenvironment. **(C)** Additional t-SNE visualization supporting the detailed identification of myeloid cell subtypes and their distinct genetic profiles, further delineating the heterogeneity within the SCCE myeloid cell population. **(D)** Comparative analysis of myeloid cell subtype distributions between naive and treatment samples, indicating shifts towards subtypes associated with increased proliferative and regulatory functions post-treatment, such as Prolif Myeloid, ProlifReg Myeloid, and CytokineReg Myeloid. This shift suggests an adaptation of the myeloid cell landscape in response to therapeutic interventions, reflecting changes in the cellular dynamics within the TME.

The t-SNE analysis of myeloid cells from SCCE samples revealed eight distinct myeloid cell subtypes, showcasing the complex heterogeneity inherent to this type of cancer. The subtypes identified include Myeloid-T helper collaborator (Mye-Th Collaborator), Replication-repair myeloid (RepliRepair Myeloid), Proliferation myeloid (Prolif Myeloid), Immune control myeloid (ImmuneControl Myeloid), Oncogenic myeloid (Onco Myeloid), Signaling myeloid (Signaling Myeloid), Proliferation regulatory myeloid (ProlifReg Myeloid), and Cytokine regulatory myeloid (CytokineReg Myeloid), as illustrated in Figures 5A, C.

KEGG pathway enrichment analysis performed on these myeloid cell clusters underscored critical pathways essential to immune responses, cell signaling, and cancer-related processes, such as cytokine-cytokine receptor interaction, Ras signaling, PI3K-Akt signaling, and NF-kappa B signaling (Figure 5B). These pathways are fundamental for the functionality of myeloid cells and their interactions within the tumor microenvironment, pivotal for immune modulation and cancer progression.

Comparative analysis between naive and treated myeloid cell subtypes demonstrated significant shifts in subtype distribution (Figure 5D). In naive samples, there was a predominance of Mye-Th Collaborator and RepliRepair Myeloid subtypes. Post-treatment, there was a noticeable increase in subtypes such as Prolif Myeloid, ProlifReg Myeloid, and CytokineReg Myeloid. These changes suggest a shift towards more proliferative and regulatory functions in response to treatment, reflecting the dynamic nature of the tumor microenvironment and its adaptive responses to therapeutic interventions.

### Dynamic interactions between tumor cells and myeloid cells in SCCE across treatment regimens

**FIGURE 6:**
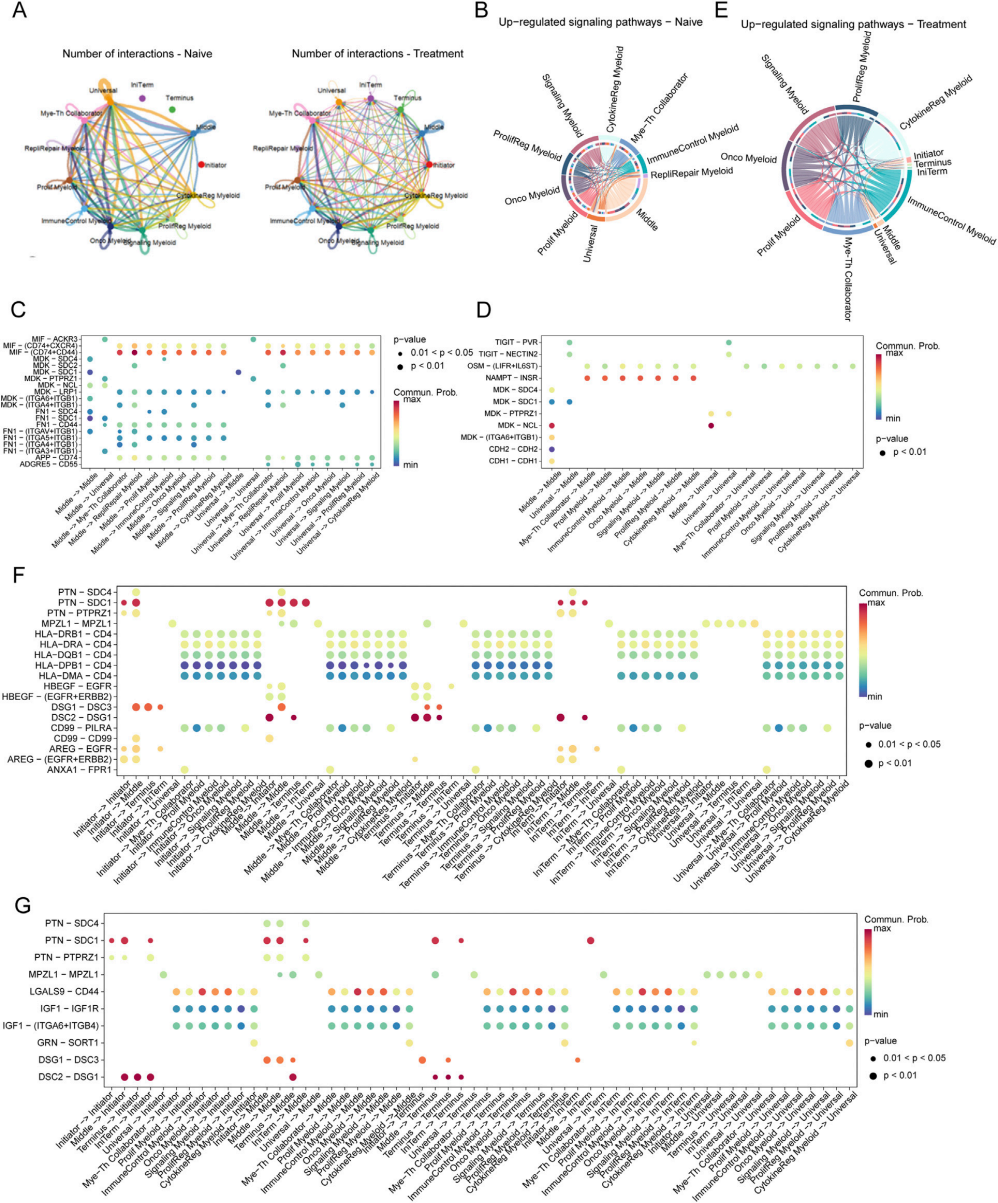
Interaction Networks and Signaling Pathways Between Tumor Cells and Myeloid Cells in SCCE Post-Treatment **(A)** Visualization of interaction networks between Initiator and Universal tumor cells and various myeloid subtypes, showing pronounced differences between naive and treatment conditions. Notable increases in interactions post-treatment highlight significant changes in cell-cell communication. **(B–D)** Pathway analysis in naive cells, demonstrating the activation of key pathways: **(B)** The MIF-CD74/CXCR4 pathway, involved in immune modulation. **(C)** The MDK-SDC1 pathway, associated with cell survival. **(D)** The HLA-E-CD8A pathway, crucial for immune suppression. **(E–G)** Pathway analysis in treated cells, depicting upregulated pathways indicative of altered cellular functions: **(E)** The TIGIT-PVR pathway, demonstrating enhanced immune checkpoint activity. **(F)** The MDK-SDC4 pathway, reflecting changes in immune checkpoint regulation. **(G)** The FN1-ITGA6/ITGB1 pathway, involved in increased cell adhesion and matrix remodeling. These pathways illustrate the dynamic responses of tumor and myeloid cells within the TME to therapeutic interventions, signifying adaptive shifts that may impact treatment efficacy and disease progression.

Analysis of the interaction networks between tumor cells and myeloid cells within the SCCE microenvironment uncovered significant insights into cellular plasticity, with notable differences between naive and treated conditions (Figure 6A). In naive samples, interactions predominantly occurred between Initiator and Universal tumor cells and various myeloid subtypes, especially the Myeloid-T helper collaborator (Mye-Th Collaborator) and Proliferation regulatory myeloid (ProlifReg Myeloid). Following treatment, a substantial increase in interactions was observed, particularly involving Initiator cells and multiple myeloid subtypes, indicating significant treatment-induced changes in cell-cell communication.

Pathway analysis further clarified the shifts in signaling pathways between conditions. In naive cells, pathways critical for immune modulation and cell survival, such as MIF-CD74/CXCR4, MDK-SDC1, and HLA-E-CD8A, were predominantly active (Figures 6B–D). Conversely, post-treatment, pathways like TIGIT-PVR, MDK-SDC4, and FN1-ITGA6/ITGB1 were significantly upregulated, suggesting enhanced immune checkpoint activity and increased cell adhesion (Figures 6E–G).

In the naive setting, substantial interactions involving Middle and Universal cells were linked to immune-suppressive pathways such as MIF-CD74/CXCR4 and HLA-E-CD8A (Figure 6C). Post-treatment, these interactions shifted to include pathways such as TIGIT-PVR and FN1-ITGA6/ITGB1, reflecting changes in immune checkpoint regulation and extracellular matrix interactions (Figure 6F).

Moreover, in naive samples, significant interactions targeted pathways such as MIF-CD74, MDK-SDC1, and HLA-E-CD8A, consistent with mechanisms of immune suppression and cell survival. In contrast, treated samples exhibited pronounced interactions involving Middle and Universal cells through pathways like TIGIT-PVR, MDK-SDC4, and FN1-ITGA6/ITGB1, illustrating an adaptive response characterized by increased immune checkpoint activity and matrix remodeling (Figure 6G).

## Discussion

**FIGURE 7:**
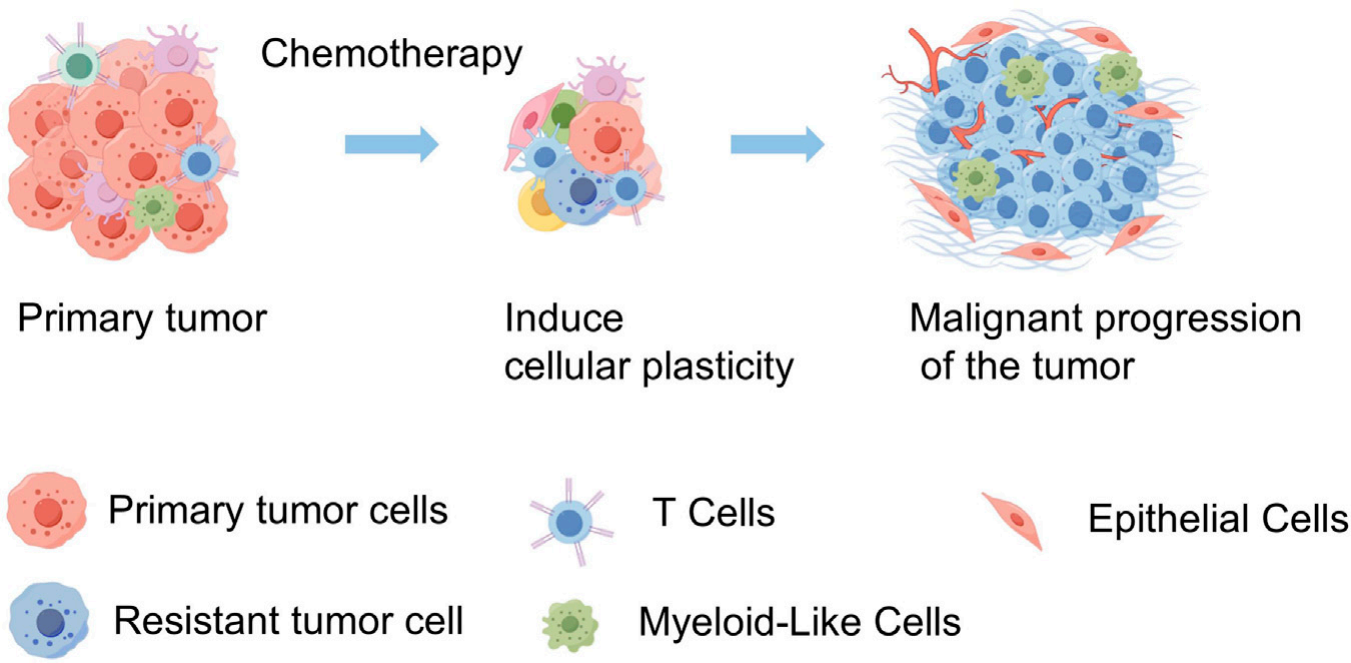
Schematic diagram of tumor progression in SCCE before and after chemotherapy. This diagram illustrates the primary tumor in SCCE, showing a reduction in tumor volume following chemotherapy. However, this treatment also induces an increase in tumor cell plasticity and heterogeneity, which contributes to the subsequent malignant progression of the tumor. This visualization highlights the dual effects of chemotherapy, underscoring the reduction in tumor size alongside the adverse enhancement of tumor complexity and potential for aggressive disease progression. (By FigDraw).

Our study employs scRNA-seq to explore the impact of chemotherapy on SCCE, by analyzing pre- and post-treatment samples from a patient diagnosed with stage III lymph node metastatic SCCE who exhibited only a partial response. Profound shifts were detected in the cellular composition of the TME. Notably, there was a significant increase in the plasticity of SCCE cells following chemotherapy—an adaptive response that likely plays a crucial role in the clinically observed rapid recurrence. These insights could inform therapeutic strategies aimed at disrupting these transitions to improve clinical outcomes. The intricate interplay within the tumor fosters a continuously evolving landscape, characterized by the coexistence of cells in both stem-like and differentiated states. This complexity complicates the efficacy of treatment and underscores the necessity for therapeutic strategies that directly address this adaptive cellular diversity (Figure 7).

To better elucidate these dynamics, we employed pseudotime analysis to map cellular transitions, identifying distinct cell states termed “Initiator” and “Terminus” (Sumanaweera et al., 2024; Sun et al., 2024). The “Initiator” state appears to represent an early adaptive response to chemotherapy, potentially harboring mechanisms associated with either initial resistance or sensitivity, while the “Terminus” state is linked to more evolved, possibly therapy-resistant phenotypes. These findings suggest that progression through these states could be pivotal in driving treatment outcomes. This enhanced plasticity aligns with the broader understanding of cancer cell plasticity, which posits that tumors leverage dynamic transitions between cell states to adapt to environmental and therapeutic pressures, contributing to both drug resistance and metastatic spread. However, it is important to note that these observations are based on a limited dataset and should be interpreted with caution. Further validation in larger cohorts is essential to confirm these preliminary findings and fully understand their implications for cancer treatment strategies.

Chemotherapy, particularly with agents such as irinotecan and cisplatin in the treatment of SCCE, profoundly influences the TME, significantly reshaping the immune landscape. Following chemotherapy, there is a notable increase in both cytotoxic and helper T cells, coupled with a decrease in exhausted T cells. This suggests a potential rejuvenation of the immune response, which is crucial for combating residual tumor cells. This reinvigoration of the immune system is further supported by pathway enrichment analyses, which reveal enhanced cytokine-cytokine receptor interactions and immune checkpoint regulation. However, this seemingly beneficial response is complicated by an increase in cellular interaction complexity, which can foster an immunosuppressive environment, thereby counteracting the initial immune gains. Additionally, the functional roles of “Initiator” and “Terminus” states may extend to the immune context, with “Initiator” cells potentially shaping initial interactions with immune cells and “Terminus” cells contributing to mechanisms of immune evasion. The intricate interplay between enhanced immune activation and the emergence of an adaptive tumor response underscores the need for integrated therapeutic strategies (Cui et al., 2024). These strategies should combine chemotherapy with targeted immunotherapies to effectively manage and treat SCCE, aiming to leverage synergistic effects to overcome the complex challenges posed by this aggressive cancer.

The interactions between tumor cells and altered myeloid cells within the TME are mediated by complex signaling pathways that become notably active following chemotherapy, particularly the TIGIT-PVR and MDK-SDC4 pathways. The TIGIT-PVR interaction is especially crucial as it plays a significant role in immune checkpoint regulation (Zhou et al., 2021). TIGIT, a T cell immunoreceptor with Ig and ITIM domains, is primarily expressed on regulatory T cells and natural killer cells (Lee, 2020; Chauvin and Zarour, 2020), and upon binding with its ligand, the PVR, expressed on myeloid cells, can inhibit T cell activation and promote immunological tolerance (Chiang and Mellman, 2022). The upregulation of this pathway post-chemotherapy suggests a strategic adaptation by the tumor to evade immune surveillance by exploiting physiological mechanisms of immune regulation (Chiang and Mellman, 2022). Recent studies have identified the TIGIT-PVR pathway as a critical axis in immune checkpoint evasion across various cancers, supporting our findings (Boissière-Michot et al., 2022; Zhou et al., 2020).

Similarly, the MDK-SDC4 pathway involves midkine (MDK), a heparin-binding growth factor, and syndecan-4 (SDC4), a transmembrane heparan sulfate proteoglycan (Hashimoto et al., 2024). This pathway is pivotal in regulating cellular proliferation, migration, and the remodeling of the extracellular matrix—processes integral to wound healing and tissue regeneration (Hashimoto et al., 2024). However, these processes may also enhance tumor invasiveness and metastasis. The increased activity of this pathway following chemotherapy indicates an escalation in extracellular matrix remodeling activities, potentially facilitating a more invasive tumor phenotype and contributing to cancer progression and spread (Cerezo-Wallis et al., 2020). Recent studies have further elaborated on the role of MDK-SDC4 in mediating the immunosuppressive environment and in the regulation of regulatory T cells in colorectal cancer development (Hashimoto et al., 2024). While these findings are compelling, their validation in larger cohorts is necessary to delineate their broader clinical implications and confirm their relevance in the context of SCCE, thereby guiding future therapeutic strategies.

This study highlights the critical need for advanced methodologies, such as single-cell proteomics and spatial transcriptomics, to achieve a refined and precise characterization of cellular dynamics within SCCE. These techniques are essential for delving deeper into the heterogeneity of cell populations, particularly to understand the dynamic changes induced by chemotherapy that enhance carcinoma cell plasticity and alter cellular interactions. Further research employing these advanced methods will facilitate the identification of patient subgroups exhibiting variable responses to chemotherapy, significantly advancing the potential for precision medicine.

## Data Availability

The datasets presented in this study can be found in online repositories. The names of the repository/repositories and accession number(s) can be found below: https://ngdc.cncb.ac.cn/gsa-human/browse/HRA008234, PRJCA028778.
