# Supplementary material for: Single-cell RNA sequencing elucidates cellular plasticity in esophageal small cell carcinoma following chemotherapy treatment
Source: Front Genet. 2025 Jan 9;15:1477705. doi: 10.3389/fgene.2024.1477705 (PMC11754407; doi:10.3389/fgene.2024.1477705)
Supplement: Supplementary file 1 [file Table1.docx]

Figure S1


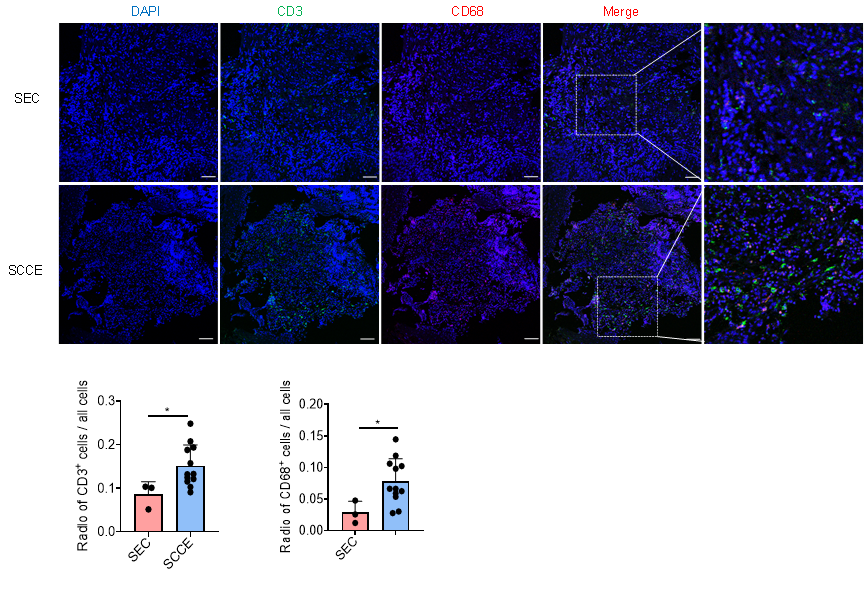


**Figure S1. Comparative Immunohistochemical Analysis of T Cells and** **Myeloid-Like Cells in Esophageal Carcinomas.** This figure presents immunostained sections from small cell carcinoma of the esophagus (SCCE; n=12) and squamous esophageal carcinoma (SEC; n=3). Tissues were probed with anti-CD3 antibodies to detect T cells and anti-CD68 antibodies to identify myeloid-like cells. Representative images illustrate the staining patterns. Scale bars: 30 μm.
